# Influence of the ANN Hyperparameters on the Forecast Accuracy of RAC’s Compressive Strength

**DOI:** 10.3390/ma16247683

**Published:** 2023-12-17

**Authors:** Talita Andrade da Costa Almeida, Emerson Felipe Felix, Carlos Manuel Andrade de Sousa, Gabriel Orquizas Mattielo Pedroso, Mariana Ferreira Benessiuti Motta, Lisiane Pereira Prado

**Affiliations:** Department of Civil Engineering, School of Science and Engineering, São Paulo State University (UNESP), Guaratinguetá 12516-410, Brazil

**Keywords:** recycled aggregate concrete, compressive strength prediction, machine learning, artificial neural networks

## Abstract

The artificial neural networks (ANNs)-based model has been used to predict the compressive strength of concrete, assisting in creating recycled aggregate concrete mixtures and reducing the environmental impact of the construction industry. Thus, the present study examines the effects of the training algorithm, topology, and activation function on the predictive accuracy of ANN when determining the compressive strength of recycled aggregate concrete. An experimental database of compressive strength with 721 samples was defined considering the literature. The database was used to train, validate, and test the ANN-based models. Altogether, 240 ANNs were trained, defined by combining three training algorithms, two activation functions, and topologies with a hidden layer containing 1–40 neurons. The ANN with a single hidden layer including 28 neurons, trained with the Levenberg–Marquardt algorithm and the hyperbolic tangent function, achieved the best level of accuracy, with a coefficient of determination equal to 0.909 and a mean absolute percentage error equal to 6.81%. Furthermore, the results show that it is crucial to avoid the use of overly complex models. Excessive neurons can lead to exceptional performance during training but poor predictive ability during testing.

## 1. Introduction

The construction industry can act in a socially antagonistic manner. While its progress contributes to economic growth and national development, the demolition of old structures caused by this industry generates a huge amount of demolished concrete in landfills and causes serious environmental problems, such as the depletion of landfill areas. To mitigate the adverse environmental effects associated with the demolition of structures, recycled aggregate concrete (RAC) from these demolitions has been used as an alternative to non-renewable natural aggregates [1,2,3,4]. In addition, the use of recycled concrete aggregate improves the sustainability of natural resources, lowers transportation costs, and reduces the CO_2_ footprint [5,6,7].

However, it should be noted that there are differences between recycled aggregate (RA) and conventional aggregate. The main difference is the higher porosity of the RA, which implies higher water absorption [8]. This effect occurs due to the amount of mortar from the original concrete adhering to the aggregate surface. The adhered mortar layer directly influences the pore structure, thereby affecting the mechanical properties of concrete made with recycled aggregate [9,10].

The compressive strength of RAC is strongly related to factors such as the water–cement ratio, the aggregate–cement ratio (both coarse and fine), the rate at which natural aggregate is replaced by RA, and the substitution rate. However, the analysis of these factors and compressive strength indicates a non-linear relationship with a certain level of complexity and, currently, there is no defined theoretical equation that can accurately reflect this behavior [11,12,13]. For practical purposes, substantial experiments are conducted to ensure that the compressive strength of RAC meets the necessary requirements, a practice that requires time and investment.

To overcome the aforementioned difficulties, the use of mathematical models has become a frequent practice for estimating the composite properties, assisting in the specification and dosage of RAC. Many studies have used statistical methods to estimate the RAC mechanical properties, relying mainly on statistical analyses involving the application of linear and non-linear regression algorithms for prediction purposes [11]. Revilla-Cuesta et al. [14] developed prediction models for the RAC’s compressive strength and elastic modulus, based on simple and multiple regressions, correlating the predictions with the composite void ratio. Younis et al. [15] statistically analyzed a dataset of RAC and, through multiple linear and non-linear regressions, established relationships between the RAC physical properties and the compressive strength. Liu et al. [16] statistically analyzed the relationships between compressive strength and different attributes, proposing a model to predict compressive strength using simple and multiple linear regression, inferring that the RA’s substitution rate and water absorption are the attributes that most influence the RAC mechanical properties.

Even with the possibility of developing models through linear and non-linear regression, simple or multiple, capturing the influence of the different materials on the composite mechanical behavior is still a costly task due to the need to determine linear or nonlinear functions capable of correlating attributes with the desired output, given the composite heterogeneity and its constituent materials. However, with the advent of artificial intelligence (AI) and machine learning techniques, it has become possible to solve problems with multiple variables, nonlinearities, and high complexity, without the need for knowledge of functions representing the relationship between the input attributes and the concrete’s compressive strength.

In recent years, the advancement of artificial intelligence has led to the creation of numerous machine learning techniques and approaches. Among these approaches, artificial neural networks (ANN) stand out for their efficiency and low computational cost, offering simple and effective solutions.

One of the reasons why ANNs are currently widely recognized and employed is related to the use of the training algorithm known as backpropagation (BP), which is responsible for adjusting the synaptic weights that connect the network neurons [17]. The BP algorithm employs a gradient descent approach to update the weight matrix but often exhibits slow convergence when dealing with complex problems. To enhance its convergence, various modifications can be applied to BP.

Due to the simplicity of implementing the BP algorithm, most studies developed and published in the civil engineering field that use ANNs opt for BP to train neural networks [18,19,20,21,22,23,24,25,26,27,28,29]. Moselhi et al. [30] were the first to investigate the application of artificial neural networks for modeling construction-related problems. They conducted an analysis of optimal real estate market conditions in different buying and selling scenarios. Following the same line of research, Chao and Skibniewski [31], Li et al. [32], and Pham et al. [33] demonstrated that ANNs can predict worker productivity in the construction sector.

Specific areas of structures and materials have been used ANN to evaluate the applicability of machine learning in civil engineering, with the majority of the studies in this field aiming to predict physical, chemical, or mechanical properties, especially in concrete. Examples include the prediction of compressive strength [26,28,29,34,35,36,37], the elastic modulus [38,39,40,41,42,43], the determination of the workability of concrete and its consistency in the fresh state [44,45,46,47], the mapping of composite degradation mechanisms [48,49,50,51,52,53,54], and the development of a concrete-mix design model [55,56,57,58].

Regarding the prediction of the RAC’s compressive strength, the use of ANN has shown great applicability and efficiency. Deshpande et al. [34] evaluated the efficiency of non-linear-regression, tree models, and ANN with a BP algorithm to predict the RAC’s compressive strength. The results indicated that the ANN models were stronger than the other techniques in terms of accuracy. In the ANN modeling, the authors considered networks with a single hidden layer, varying the number of neurons in this layer. The optimal number of neurons in the hidden layers was determined based on the training performance. All the networks were trained using the Levenberg–Marquardt algorithm with the ‘log–sigmoid’ transfer functions between the first (input) and second (hidden) layers and a ‘linear’ transfer function between the second and third layers (output). The network with the best performance was generated with ten inputs, one output, and 32 neurons in the hidden layer, achieving a determination coefficient (*R*^2^) of 0.9025 and an average absolute relative error percentage of 9.31%.

Duan et al. [35] studied ANNs’ applicability to predicting the compressive strength of RAC. The models were developed considering a database with 146 experimental data, and training was conducted using the backpropagation-momentum training algorithm, a variant of stochastic gradient descent. Different topologies were evaluated, considering 14 neurons in the input layer, one in the output layer, and up to two hidden layers, each containing 5 to 50 neurons. During the training, the authors tested different values for the learning rate (0.01, 0.1, 0.3, 0.5, 0.7, 0.9, 1.0, and 2.0) and the momentum factor (0.0, 0.3, 0.5, 0.7, 0.9, and 1). Based on the error obtained in a testing step after a training series, the best topologies and parameters that maximized the *R*^2^ values were one hidden layer with sixteen neurons, a momentum variable equal to 0.9, and a learning rate equal to 0.3.

Rizvon et al. [59] evaluated the applicability of three machine learning techniques for predicting the RAC’s compressive strength: random forest, ANN, and least absolute shrinkage and selection operator (LASSO). The networks were trained using a single hidden layer, seven neurons in the input layer, and a single output. The Levenberg–Marquardt algorithm was used in the ANN training. The technique with the best performance was determined with 12 neurons in the hidden layer, achieving a determination coefficient of 0.92 and an AARE of 2.54%.

Bu et al. [29] applied artificial neural networks with the Levenberg–Marquardt training algorithm to predict the RAC’s compressive strength. A dataset comprising 88 data points was acquired through comparative tests involving various mixed-proportion designs. This dataset was used to create an ANN, whose optimal architecture was determined through a trial–error approach, considering two hidden layers with 1–20 neurons. The input parameters considered in this process included the following: cement content, sand content, natural coarse aggregate content, recycled coarse aggregate content, water content, water–colloid ratio, sand content rate, and the recycled-aggregate-replacement rate. As activation functions, the authors used two sigmoid functions, the log–sigmoid and the tan–sigmoid. The findings indicate that the most effective ANN architecture comprised eight input neurons, 12 neurons in the hidden layer, and eight output neurons, leading to a single output neuron. This configuration achieved a notable determination coefficient of 0.96. The root mean square error was recorded at 2.42 MPa, and the chosen activation function was the logistic sigmoid.

Based on results in the literature, artificial neural networks are potential tools for predicting the compressive strength of concrete, assisting in the RAC mix design and, thus, contributing to the environmental impact reduction generated by the construction industry. However, due to the material’s heterogeneity, the number of parameters related to mechanical properties, different training algorithms, and the ANN-training configuration, most of the works in the literature do not clearly present the methodology adopted in the search for the hyperparameters that configure the best network learning; in many cases, this search is performed randomly. Generally, a random process for determining accurate models can become a costly process, requiring time and effort to find the configuration that generates the best machine learning.

Thus, the main purpose of this study is to examine how the topology, ANN-training algorithm, and activation function affect the learning and performance of the RAC’s compressive strength prediction model developed by feedforward ANN with backpropagation algorithms. This study uses a diverse database (consisting of 721 data samples) comprising the experimental results of the RAC’s compressive strength, including the aggregate from cementitious material waste obtained from various research groups and collected from 38 literature works [2,8,9,35,42,60,61,62,63,64,65,66,67,68,69,70,71,72,73,74,75,76,77,78,79,80,81,82,83,84,85,86,87,88,89,90,91,92]. This study combines three training algorithms (backpropagation Momentum, backpropagation Delta–Bar–Delta, and Levenberg–Marquardt), two activation functions (hyperbolic tangent and logistic sigmoid), and 40 topologies (networks with a hidden layer containing 1 to 40 neurons). After training and analyzing the 240 ANNs, the results provide valuable insights into defining the optimal training hyperparameters. The findings will help to reduce the time spent searching for the best topologies and algorithms that yield the best learning and prediction accuracy.

## 2. Artificial Neural Networks

Artificial neural networks are parallel and distributed systems that use processing units like biological neural networks. One of the main aspects of these networks is that they can map and represent complexity and nonlinear problems [93].

**Figure 1:**
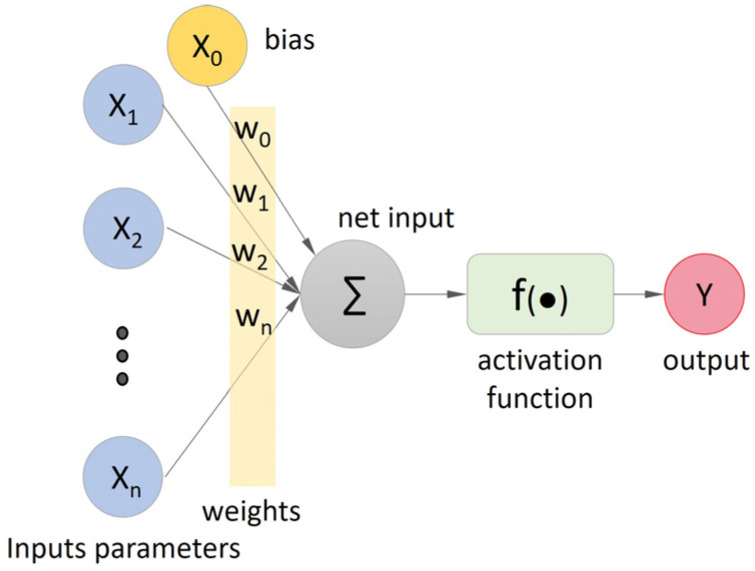
Representation of a perceptron neural network.

Inspired by the human brain, artificial neural networks establish connections with neurons, organizing them into one or more layers. These connections are characterized by synaptic weights, which can be used to store information. Haykin [94] states that artificial neural networks have five essential elements, as in the perceptron neural network illustrated in Figure 1. These elements are associated with: (i) a set of inputs, each with its weight, simulating dendritic activity; (ii) an adder processing input signals through neuron synapses; (iii) an activation function constraining the output range; (iv) a bias adjusting the net input to the activation function; and (v) a network-generated output symbolizing the axonal response. Thus, Equation (1) represents a single perceptron with n inputs.

(1) $$y=f\left(\sum_{i=1}^{n}w_{i}x_{i}+b\right)$$

where each input $x_{i}\in R$, with i=1,…,n, is weighted by a corresponding $w_{i}\in R$, which forms the current neuron weight vector $w={\left(w_{1},\ldots ,w_{n}\right)}^{T}$. The bias b∈R is a permanent internal addition that represents the model vies. The f is an activation function that takes the net input from the sum function and produces the neuron output, y∈R.

### 2.1. Multilayer-Perceptron Neural Networks

**Figure 2:**
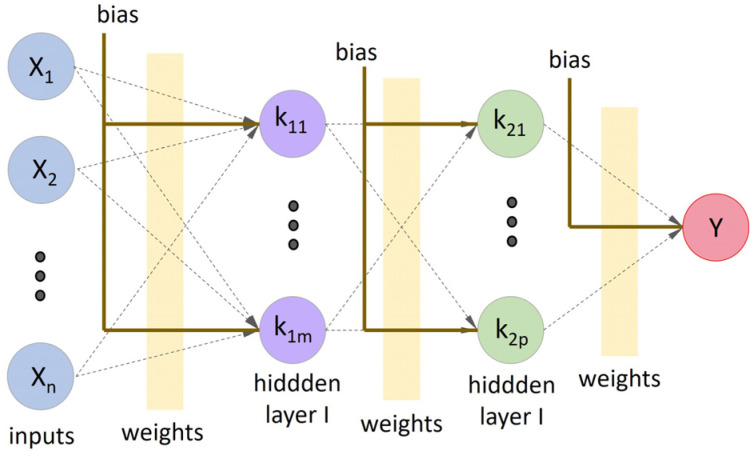
Representation of an MLP ANN with two hidden layers.

Multilayer-perceptron neural networks (MLP) support multiple processing neurons and layers (Figure 2), allowing models to solve nonlinear problems.

The MLP comprises interconnected layers—input, hidden, and output. The input layer processes features, whereas the hidden layers use weighted sums and activation functions to introduce nonlinearity [94]. Using an appropriate activation function, the output layer generates predictions. During training, a feedforward network adjusts weights through a backpropagation algorithm to minimize prediction errors. The weights are fine-tuned using optimization algorithms, such as gradient descent.

### 2.2. Training Algorithm

In a supervised learning environment, backpropagation is a crucial algorithm for training ANN, specifically the multilayer-perceptron type. The approach begins with a forward pass, during which input data are propagated through the network to compute the projected output. This includes computing the output of each neuron using a weighted sum of inputs and an activation function. The error is then computed by comparing the predicted output to the target output using a suitable loss function.

The algorithm then moves backward through the network iteratively to compute the error gradient associated with weights, which is the most critical backpropagation training stage. This gradient indicates the direction in which the weights must be modified to reduce the error. Subsequently, the weights are adjusted in the opposite direction to the gradient using an optimization process, often gradient descent. Most learning–training algorithms use the same calculation routine, with the only difference being the method for adjusting the synaptic weights. Backpropagation classic (BPC) uses Equations (2) and (3) to change the synaptic weights at each iteration.

(2) $${\Delta w}_{i,j}^{\left[k\right]}={\alpha \delta }_{i,j}^{\left[k\right]}y_{i,j}^{\left[k\right]}$$

(3) $$w_{i,j}^{\left[k+1\right]}=w_{i,j}^{\left[k\right]}+{\Delta w}_{i,j}^{\left[k\right]}$$

where ${\Delta w}_{i,j}^{\left[k\right]}$ represents the neuron’s synaptic weight update *i* in layer *j*, α is the learning rate, $\delta_{i,j}^{\left[k\right]}$ is the error associated, $y_{i,j}^{\left[k\right]}$ is the predicted value, and $w_{i,j}^{\left[k+1\right]}$ is the synaptic weight’s updated value.

The learning rate (α) determines each weight update’s magnitude, guaranteeing that the optimization process converges [94]. This iterative approach is performed for multiple epochs until the error is acceptable, training the neural network to predict accurately. This pace is constant throughout the processing layer and with each BPC iteration (network processing step, index k). Thus, in classic backpropagation, α is a fixed hyperparameter that is set at the start of the training.

Through the inclusion of an impulse filter (the momentum variable) in the weight correction, a heuristic modification transforms BPC into backpropagation momentum (BPM) [95]. In Equation (3), a weight adjustment known as the Momentum rate (µ) is included, generating Equation (4).

(4) $$w_{i,j}^{\left[k+1\right]}=w_{i,j}^{\left[k\right]}+µ{\Delta w}_{i,j}^{\left[k-1\right]}+\left(1-µ\right){\Delta w}_{i,j}^{\left[k\right]}$$

Note that the BPM is reduced to BPC if µ = 0. As a result, it is commonly stated that the variable must be defined within the interval (0, 1] for the BPM method to have an effect and enable network-convergence optimization. The learning rate remains constant, but the weights of two consecutive iterations must be stored [94].

Backpropagation delta–bar–delta (BPD) alters backpropagation classic heuristically, allowing the learning rate to perform dynamically [95]. This rate is governed by the gradient descent. The increment signal is the partial derivative of the error function concerning the synaptic weight. If the weights of two iterations are in the same direction, the gradient is in the direction of the local minima. The learning rate then increases when the weights are in opposite directions and decreases when they are in the same direction. The algorithm changes are shown in Equations (5) and (6).

(5) $$\alpha_{i,j}^{\left[k+1\right]}=\left\{\begin{array}{c} \alpha_{i,j}^{\left[k\right]}+\varphi ,\;\;\;se\;\left[\frac{\partial E}{\partial w_{i,j}^{\left[k-1\right]}}\right]\left[\frac{\partial E}{\partial w_{i,j}^{\left[k\right]}}\right]>0,\\ {(1-\gamma )\alpha }_{i,j}^{\left[k\right]},\;\;\;se\;\left[\frac{\partial E}{\partial w_{i,j}^{\left[k-1\right]}}\right]\left[\frac{\partial E}{\partial w_{i,j}^{\left[k\right]}}\right]<0,\\ \alpha_{i,j}^{\left[k\right]}\;\;\;se\;\left[\frac{\partial E}{\partial w_{i,j}^{\left[k-1\right]}}\right]\left[\frac{\partial E}{\partial w_{i,j}^{\left[k\right]}}\right]=0. \end{array}\right.$$

(6) $$w_{i,j}^{\left[k+1\right]}=w_{i,j}^{\left[k\right]}-\alpha_{i,j}^{\left[k+1\right]}\frac{\partial E}{\partial w_{i,j}^{\left[k\right]}}$$

where γ and φ are constants set at the start of training within the interval (0,1), *E* is the error function, and $\frac{\partial E}{\partial w_{i,j}^{\left[k\right]}}$ represents the derivative in terms of the synaptic weight.

The Levenberg–Marquardt (LM) training algorithm is a well-known optimization method for training artificial neural networks. It is especially useful for non-linear least-squares problems, such as those encountered in neural network training, where the goal is to minimize the difference between predicted and actual output values [96]. The LM algorithm, developed as an extension of the Gauss–Newton method, dynamically combines features of both the steepest descent (gradient descent) and Gauss–Newton algorithms, effectively adapting the optimization problem’s characteristics [97].

The LM algorithm, in essence, adds a damping factor to the Gauss–Newton method, which aids in controlling the step size during weight updates. Based on the current progress, this damping factor is adjusted iteratively during the training process. In regions where the model is poorly conditioned, the damping factor is increased to avoid large steps that could exceed the minimum, like how the steepest descent algorithm behaves. As a result, the Levenberg–Marquardt algorithm seeks synaptic weights that reduce the network error. Equation (8) depicts Newton’s iterative process of pursuing weights that leads Equation (7) to a minimum, where $t_{l}$ is the real answers to the problem, $y_{l}$ is the network output, *l* is the number of data used in neural training, and ∇E and $\nabla^{2}E$ are the gradient and hessian matrix of the error function *E*, respectively.

(7) $$E=\sum_{l=0}^{L-1}(t_{l}{-y_{l})}^{T}\left(t_{l}-y_{l}\right)$$

(8) $${\left(t_{l}-y_{l}\right)}^{\left[k+1\right]}={\left(t_{l}-y_{l}\right)}^{\left[k\right]}-\left[\nabla^{2}E\left(x\right){|}_{x={t_{l}-y_{l}}^{\left[k\right]}}\right]\left[\nabla E\left(x\right){|}_{x={t_{l}-y_{l}}^{\left[k\right]}}\right]$$

### 2.3. Activation Function

By considering the neuron’s intrinsic state, the activation function (as shown in Figure 1) converts the net input signal into the net output [94]. Numerous activation functions are available. Felix et al. [22] state that the most frequently used activation functions in the ANN training to map the concrete mechanical properties are linear, stepped, logistic sigmoid (LOG-SIGMOID), and the hyperbolic tangent (TANH), in Equations (9)–(12), respectively. In most relevant studies, in regression mappings, these activation functions are only applied at the output layer, which processes the outputs. The rectified linear unit function (ReLU), shown in Equation (13), is frequently employed in hidden layers.

(9) f(x)=x

(10) $$f\left(x\right)=\left\{\begin{array}{c} 0,\;\;\;\;x<0\\ 1,\;\;\;\;x\geq 0 \end{array}\right.$$

(11) $$f(x)=\frac{1}{1+e^{-x}}$$

(12) $$f(x)=\frac{\left(e^{x}-e^{-x}\right)}{\left(e^{x}+e^{-x}\right)}$$

(13) $$f(x)=max\left(0,\;x\right)$$

The RAC’s compressive strength was recently mapped by Bu et al. [29] using artificial neural networks and the Levenberg–Marquardt training algorithm, testing various activation functions. The results show that the logistic sigmoid function performed the best. Furthermore, Felix et al. [38] investigated the effectiveness of logistic sigmoid and hyperbolic tangent functions in mapping the RAC elastic modulus. The findings suggested that using the hyperbolic tangent function can improve prediction accuracy.

According to Haykin [94], sigmoid functions are the most frequently used functions in ANN training. This preference stems from their homogeneity, asymptotic nature, continuity, symmetry, monotonic increasing behavior, limitations, and the ease of obtaining derivatives.

## 3. Methodology

**Figure 3:**
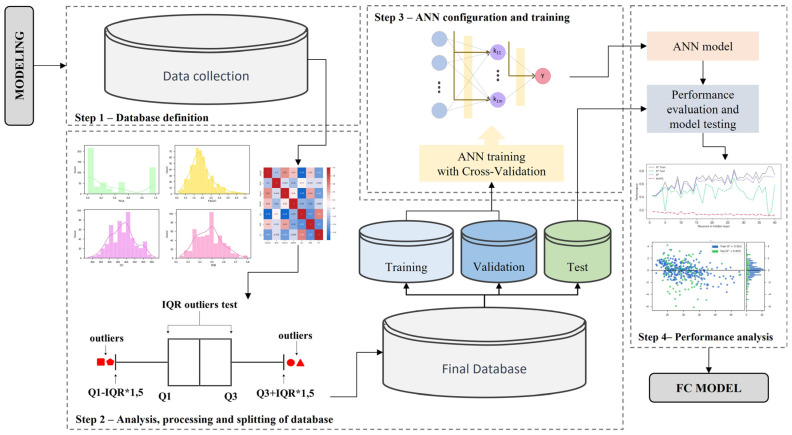
Flowchart of the methodology employed in the ANN modeling.

The methodological procedure depicted in Figure 3 was used in this study to generate artificial neural network regression models to predict the compression strength of concrete-containing recycled aggregates. Different topologies, training algorithms, and activation functions were combined to create the ANN models. The modeling procedure was divided into four stages, which are described in detail in the following sections.

### 3.1. Data Definition

**Figure 4:**
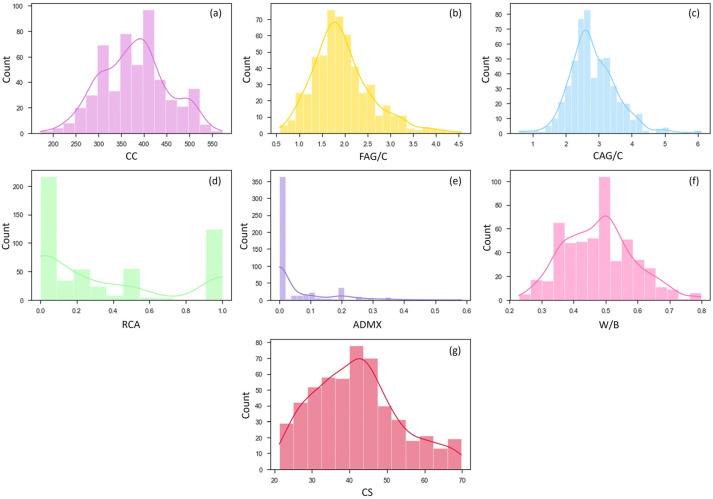
Distribution of data as a function of (**a**) CC, (**b**) FAG/C, (**c**) CAG/C, (**d**) RCA, (**e**) ADMX, (**f**) W/B, and (**g**) CS.

The results of experimental compression-strength tests at 28 days (CS) performed by various research groups were collected from 38 studies [2,8,9,35,42,60,61,62,63,64,65,66,67,68,69,70,71,72,73,74,75,76,77,78,79,80,81,82,83,84,85,86,87,88,89,90,91,92], forming the initial database, which contained 721 samples. The initial database was created using results in the literature for concrete with recycled coarse aggregate derived from cementitious waste (concrete and mortar). Cement consumption in kg/m^3^ (CC), fine-aggregate–cement ratio (FAG/C), coarse-aggregate–cement ratio (CAG/C), recycled-aggregate-replacement rate (RCA), mineral-admixture–cement ratio (ADMX), and water–binder ratio (W/B) were the features of the collected data. Figure 4 depicts the frequency distribution of the entire database based on the features, and Table 1 displays the maximum, minimum, average, standard deviation, and first-, second-, and third-quartile values.

### 3.2. Database Analysis, Processing

Some observations within a dataset may be beyond the overall scope of other observations. Outliers are observations that deviate from the norm. On the original dataset, the inter quartile range (*IQR*) outlier-detection approach was employed to examine the database’s representativeness and discover any outliers. The *IQR* is calculated by the difference between the third (*Q*3) and first (*Q*1) quartiles, as indicated in Equation (14). After computing the *IQR*, it is advisable to remove data from the database that have attributes with values that are either above the upper limit (*UL*) or below the lower limit (*LL*), both of which are calculated by Equations (15) and (16), respectively.

(14) IQR=Q3−Q1

(15) UL=Q3+1.5(QR)

(16) LL=Q1−1.5(IQR)

**Figure 5:**
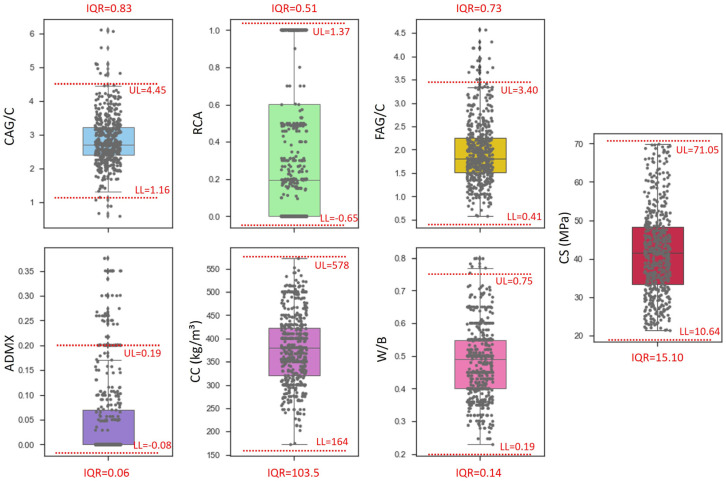
Data distribution, QR, upper and lower limits defined with IQR outlier-detection method.

Figure 5 depicts a data distribution boxplot with respect to the input features and compressive strength, as well as the *IQR*, *UL*, and *LL*. Data that exceeded the upper and lower limits were removed, reducing the initial database to 627 data.

After removing the outliers from the database, it was divided into three subsets: a training dataset with 70% of the data, a validation dataset with 15%, and a test dataset with 15%. The three subsets were defined using the stratified sampling approach, which ensured that all subsets had the same proportions as the database and that each grouping of interest was represented.

### 3.3. ANN Configuration and Training

**Figure 6:**
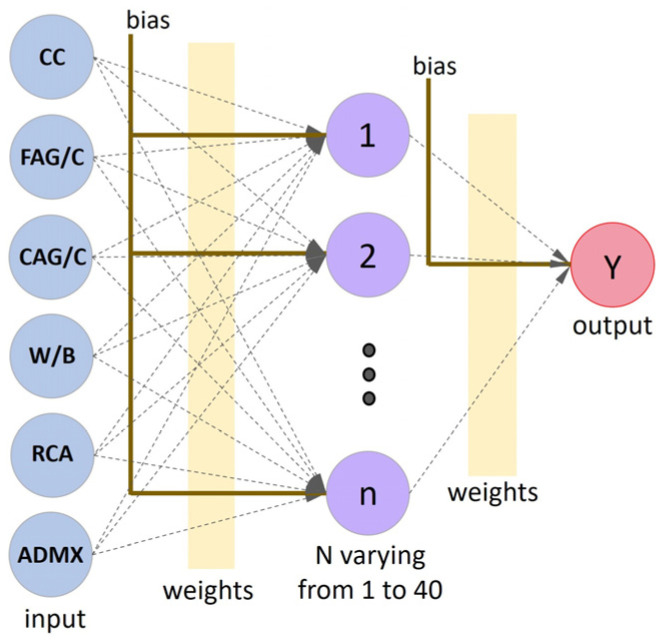
The typical ANN topology employed in the modeling process.

Forty ANN topologies were designed to predict the RAC’s compression strength, with six neurons in the input layer (associated with CC, FAG/C, CAG/C, W/B, RCA, and ADMX), a hidden layer with neurons ranging from 1 to 40, and one neuron in the output layer (Figure 6). The ANNs were trained using the cross-validation method, as suggested by Felix et al. [22].

Backpropagation Delta–Bar–Delta (BPD), backpropagation Momentum (BPM), and Levenberg–Marquardt (LM) algorithms were used to train the ANN. The logistic sigmoid (LOG-SIGMOID) and hyperbolic tangent (TANH) functions were used to calculate the output layer throughout the training procedure. The ReLu function was applied to calculate the signals associated with the input layer.

The mean square error (MSE), presented in Equation (17), was established as a training convergence criterion. A maximum of 10^6^ iterations was allowed.

(17) $$MSE=\sum_{i=1}^{n}\frac{{\left(y_{i}-t_{i}\right)}^{2}}{n}$$

in which $t_{i}$ is the observed values, $y_{i}$ represents the predicted outputs, and n is the amount of data evaluated.

To analyze the influence of topology, algorithm training, and activation function on all ANN training, the initial weights were set to 0.5. Based on Duan et al. [35], the learning rate and momentum variable have initial values of 0.3 and 0.9, respectively. The code was written in Python because of its ease of integration with modules specialized to data manipulation, analysis, and display.

### 3.4. Performance Analysis

Several metrics are used in machine learning regression to evaluate models’ performance in predicting continuous parameters. To measure the performances of all ANNs trained in this study, the root-mean-square error (*RMSE*), mean absolute percentage error (MAPE), and coefficient of determination (*R*^2^) were calculated.

The root-mean-square error, presented in Equation (18), is used to calculate the average size of errors while giving more weight to larger errors, making it appropriate for circumstances in which large errors are more critical or destructive. The *RMSE* is useful because it quantifies the average prediction error and highlights significant errors’ importance by squaring the differences.

(18) $$RMSE=\sqrt{\sum_{i=1}^{n}\frac{{\left(y_{i}-t_{i}\right)}^{2}}{n}}$$

The determination coefficient, presented in Equation (19), sometimes known as R-squared, is a statistic used in regression analysis to assess how well a regression model fits the observed data. The *R*^2^ expresses how much of the variance in the dependent variable can be predicted by model’s independent variables.

(19) $$R^{2}={\left(\frac{\sum_{i=0}^{I-1}\left(t_{i}-\bar{t}\right)\left(y_{i}-\bar{y}\right)}{\sqrt{\sum_{i=0}^{I-1}{\left(t_{i}-\bar{t}\right)}^{2}{\left(y_{i}-\bar{y}\right)}^{2}}}\right)}^{2}$$

The mean absolute percentage error, presented in Equation (20), is stated in percentage terms, making it simple to understand. When compared to other error metrics, such as *MSE* or *RMSE*, *MAPE* is less sensitive to outliers. It provides a more balanced perspective on forecast accuracy, particularly in cases where high values may have a significant influence on other error measures.

(20) $$MAPE=\frac{1}{n}\sum_{i=1}^{n}\left|\frac{t_{i}-y_{i}}{t_{i}}\right|$$

## 4. Results and Discussion

The performance metrics *R*^2^, *RMSE*, and *MAPE* were examined after training all 240 ANNs, defined by combining the three training methods (BPM, BPD, and LM) with the two activation functions (TANH and LOG-SIGMOID) and with the forty topologies (Figure 6).

**Figure 7:**
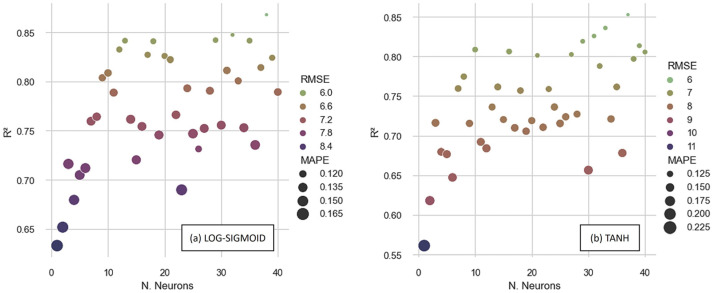
Performances of ANNs trained with BPM with (**a**) LOG-SIGMOID and (**b**) TANH functions.

Figure 7 shows the performance metrics obtained by analyzing the entire database, considering the artificial neural networks trained with the BPM algorithm. When analyzing the determination coefficients, the best performance obtained with the LOG-SIGMOID was 0.867 with a topology of [6-38-1]. The ANN architecture is denoted in this work as [x-y-z], where x is the number of input features (which we fixed in 6), y is the number of neurons in the hidden layer (ranging from 1 to 40), and z is the number of output neurons (fixed in 1). 

When using the TANH activation function, [6-37-1] generates the highest overall determination coefficient, 0.852. In terms of error metrics, the [6-38-1] with the LOG-SIGMOID function and [6-37-1] with the TANH function had the lowest *MAPE* and *RMSE* for each activation function. Thus, when the BPM method was used, the [6-38-1] using the LOG-SIGMOID function produced the best results, with the *RMSE* being 5.61 MPa and the *MAPE* being 10.56%.

**Figure 8:**
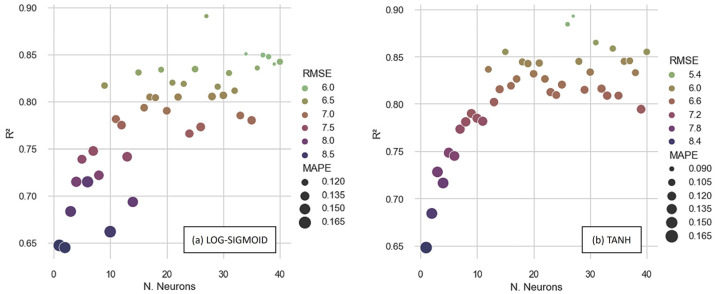
Performance of ANNs trained with BPD with (**a**) LOG-SIGMOID and (**b**) TANH functions.

Figure 8 depicts the performance metrics produced by examining the full database while using artificial neural networks trained with the BPD algorithm. When evaluating the determination coefficients, the best performance obtained using the LOG-SIGMOID function was 0.891, with the architecture [6-27-1]. The [6-27-1] configuration provided the highest determination coefficient, 0.894, when training with the TANH function. 

**Figure 9:**
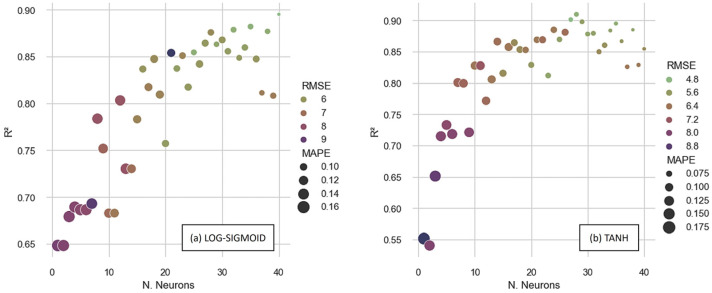
Performances of ANNs trained with LM with (**a**) LOG-SIGMOID and (**b**) TANH functions.

Using the TANH function, the topology [6-27-1] also achieved the lowest *RMSE* (5.09 MPa) and *MAPE* (8.89%). However, when evaluating the error metrics obtained with the configuration that generated the best *R^2,^* with the ANN that used the LOG-SIGMOID, the [6-27-1] did not exhibit the smallest values. Instead, the [6-39-1] achieved the lowest *RMSE* (5.98 MPa) and *MAPE* (11.96%), with a *R*^2^ of 0.852. Consequently, the outcomes depicted in Figure 9 indicate that [6-37-1], utilizing the BPD algorithm, yielded the model with the best performance, specifically when employing the TANH activation function. 

Figure 9 depicts performance metrics produced by ANNs trained with the LM algorithm. When the determination coefficients were evaluated, the optimal performance was obtained using the LOG-SIGMOID function, and the value was 0.894, with the architecture [6-40-1]. This topology also produced the lowest error metrics, with an *RMSE* of 5.04 MPa and a *MAPE* of 8.01%. 

When utilizing the TANH function, the [6-28-1] provided the highest determination coefficient, reaching 0.909. However, when the error metrics were evaluated, the [6-28-1] did not generate the lowest *MAPE*, equal to 6.56%, which was attained by the ANN [6-38-1]. The *RMSE* of the [6-38-1] was 4.69 MPa, while the *R*^2^ was 0.884. These results highlight that the best performance was demonstrated by the ANN trained with the TANH function. However, based simply on an examination of Figure 9b, it was not possible to select the topology with the best performance, since while [6-28-1] scored the highest *R*^2^, the ANN [6-38-1] provided the lowest error metrics.

**Figure 10:**
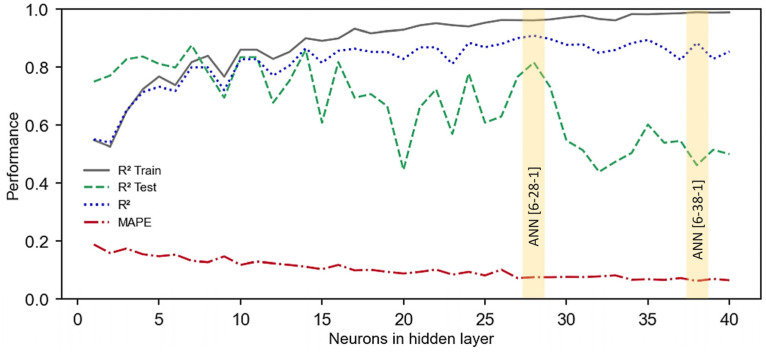
Performances of ANNs trained with LM algorithm and the TANH function.

Figure 9b shows that the network with the best overall determination coefficient, determined by analyzing all the databases, may not always produce the lowest errors. This occurs because the performance in training tends to be stronger than the performance in test phase. To evaluate this case, the *R*^2^ produced in the training (*R*^2^ Train) and testing (*R*^2^ Test) steps and the *MAPE* values were analyzed and presented in Figure 10.

Figure 10 illustrates that when the number of neurons in the hidden layer exceeds 28, the ANN performance in the training phase improves but declines in the testing analysis. This results in the creation of rigid models, in a process known as overfitting. Rigid models fit training data well but lack predictive power for new predictions [98]. Therefore, since the aim is to produce predictive models that accurately depict real conditions, the topology [6-28-1] demonstrates superior performance in comparison to [6-38-1]. This is because it possesses a higher determination coefficient in testing (0.836), with similar values for the *MAPE* and overall *R*^2^.

**Figure 11:**
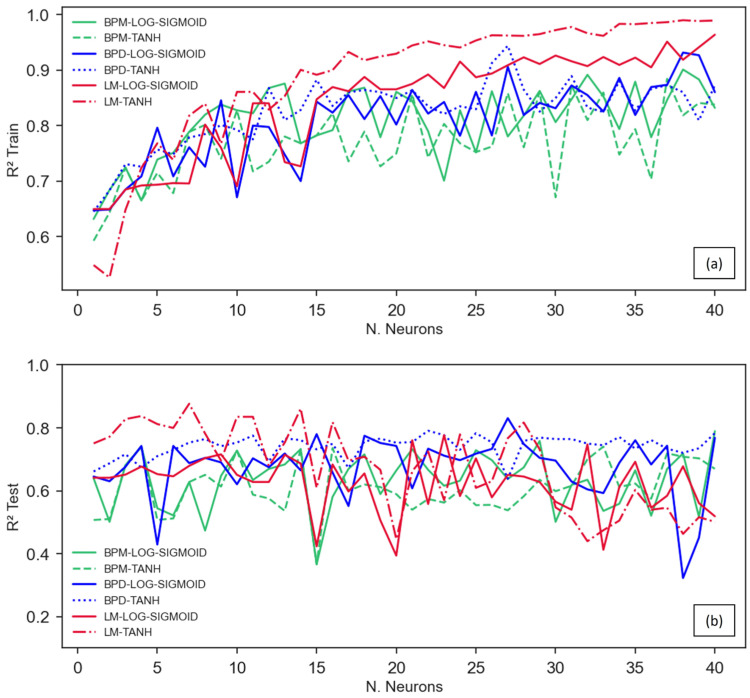
Analysis of the *R*^2^ obtained in (**a**) training and (**b**) testing phases.

**Figure 12:**
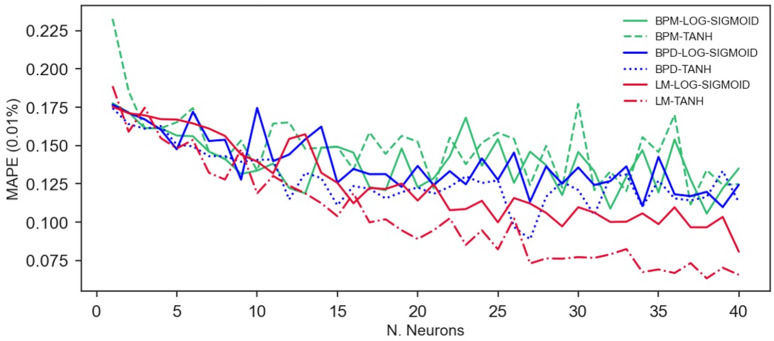
*MAPE* variation with the number of neurons, activation function, and algorithm.

A noticeable pattern can be observed in Figure 7, Figure 8 and Figure 9, where the TANH function consistently demonstrates a stronger performance in determining the RAC’s compressive strength, especially when the BPD and LM algorithms are utilized. This pattern is depicted in Figure 11 and Figure 12, which also demonstrates that the TANH function improved the ANNs’ learning and model’s generalization at a rate of 87%.

The findings presented in Figure 11 and Figure 12 indicate that the Levenberg–Marquardt algorithm generated more accurate predictions with smaller errors and greater adjustments. From Figure 11a, it can be inferred that there is no influence of the training algorithm on the ANNs’ learning with up to twelve neurons in the hidden layer. In contrast, for all the ANNs, the utilization of LM resulted in superior performance when applying more than 12 neurons in the hidden layer, with the BPD and BPM algorithms following suit. An analogous point can be made with respect to the *MAPE*, as illustrated in Figure 12. In contrast, it is worth noting that both the BPD and the LM achieved comparable performance levels when evaluating the ANNs’ generalization potentiality, considering the *R*^2^ test (Figure 11b).

**Figure 13:**
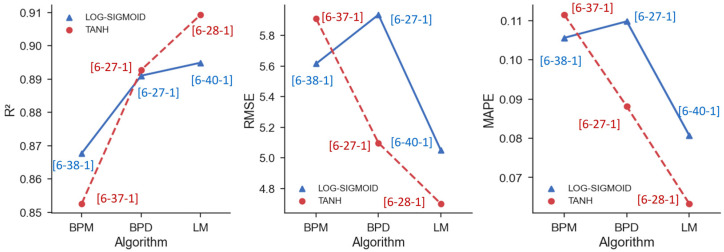
Best performances in all training scenarios.

To facilitate the comparison between the ANNs’ overall performance and the optimal performances for each of the six modeling scenarios (defined by the combination of two activation functions with three training algorithms), the determination coefficient, *RMSE*, and *MAPE* values are presented in Figure 13. 

The findings illustrated in Figure 13 provide further evidence of the LM algorithm’s superior performance. Furthermore, improved learning and greater precision were achieved by utilizing the hyperbolic tangent activation function with the LM and BPD functions. Upon evaluating the topologies that achieved the best results in each scenario, it became evident that the ANNs with a single hidden layer tended to exhibit better performances when using 27–40 neurons, independently of the algorithm used. 

**Figure 14:**
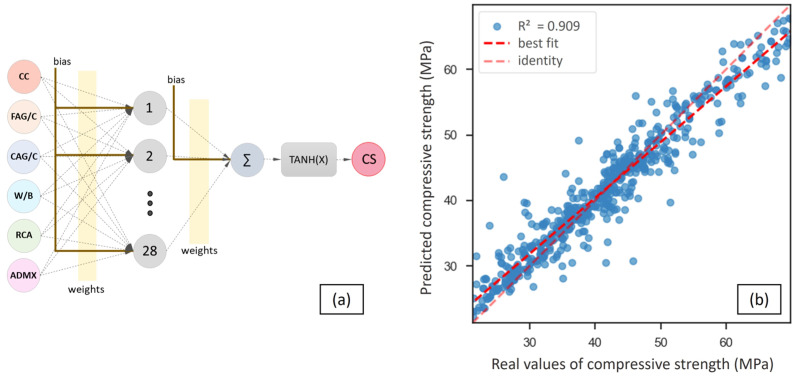
ANN with the best accuracy: (**a**) topology and (**b**) prediction performance.

Deshpande et al. [34] trained an ANN with the LM algorithm to predict the RAC’s compressive strength, and the best performance topology was generated with ten inputs, one output, and 32 neurons in the hidden layer, achieving an *R*^2^ of 0.902 and a *MAPE* of 9.31%. In this work, the ANN with the topology of [6-28-40], trained using the Levenberg–Marquardt algorithm and employing the hyperbolic tangent activation function, demonstrated the highest accuracy, boasting a training *R*^2^ of 0.909, an *RMSE* of 4.69 MPa, and a *MAPE* of 6.81%. The ANN model with the best performance is illustrated in Figure 14, which also includes a scatter plot of the compressive strength’s predicted and real values.

**Figure 15:**
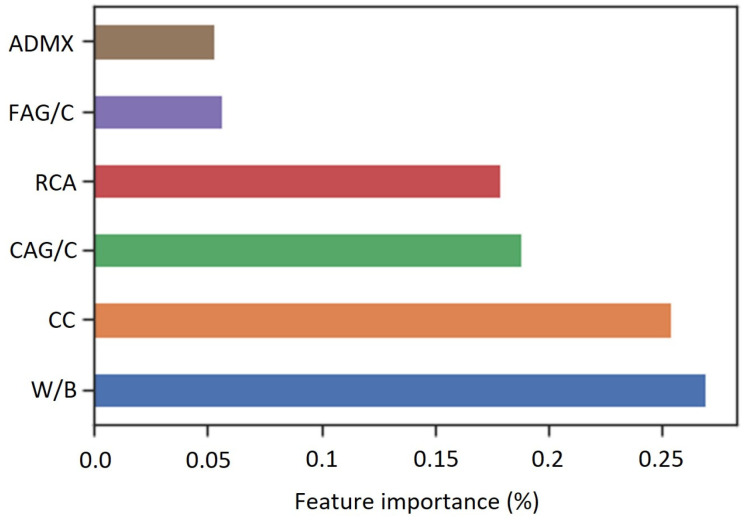
Input features’ influence on the prediction with the ANN model.

The widespread adoption of machine learning models can be attributed to their capacity to effectively capture complex, nonlinear data relationships, enabling the creation of robust forecasting formulations. However, the lack of transparency in ANN models sometimes results in limited interpretability and the inability to quantify the impact of input factors on the output. To overcome this issue, a sensitivity analysis evaluating the input features’ significance was performed, as shown in Figure 15. 

The feature importance was calculated utilizing the method presented by Milne [99], which is based on synaptic weights, as shown by Equation (21), and does not make any assumptions about the nature of the data.

(21) $$Feature\;Importance=\frac{\sum_{k=1}^{n_{hidden}}\frac{w_{ji}}{\sum_{l=1}^{n_{feature}}\left|w_{jl}\right|}w_{oj}}{\sum_{k=1}^{n_{feature}}\left(\sum_{j=1}^{n_{hidden}}\left|\frac{w_{jk}}{\sum_{l-1}^{n_{feature}}\left|w_{jl}\right|}w_{oj}\right|\right)}$$

in which *w_ji_* is a synaptic weight that connects input and hidden layers, *w_oj_* a is synaptic weight that connects hidden and output layers, *l*, *j*, and *k* refer to the input layer neuron, *n_feature_* is the input neurons number, and *n_hidden_* is the number of neurons in hidden layer.

From Figure 15, it can be observed that the ANN-based model is significantly influenced by the RCA (17.7%), CAG/C (18.8%), CC (25.3%), and W/B (26.6%). These results are consistent with the results reported by Bu et al. [29], who demonstrated that the accuracy of the predictions of their proposed ANN model was significantly affected by the cement concentration. Furthermore, the feature importance of the RCA calculated by Bu et al. [29] was 18.79%, which is very close to the value obtained in this study, which used a different database. This indicates that the recycled aggregate’s replacement rate has an influence of 18% on the accuracy of the prediction of the RAC’s compressive strength, independently of the dataset utilized in the training stage.

The ADMX and FAG/C characteristics had little impact on the accuracy of the prediction of the RAC’s compressive strength, with each having an impact of up to 6%. These results indicate that all six input features utilized in the model made a significant contribution to the ANN learning and on the accuracy of the prediction once the importance features were greater than two percent, a threshold value defined in [29].

## 5. Conclusions

An investigation was conducted to evaluate the performance of artificial neural networks in predicting the compressive strength of recycled aggregate concrete. A total of forty topologies were created and trained using three different training algorithms (backpropagation Momentum, backpropagation Delta–Bar–Delta, and Levenberg–Marquardt), and two output activation functions (hyperbolic tangent and logistic sigmoid) were considered to create and train 240 ANNs. The results of both the training and the testing phases were analyzed to draw the following conclusions:

- Activation-function influence: consistency was observed with the hyperbolic tangent’s activation function, consistently showcasing a stronger performance in predicting the compressive strength, especially under the BPD and LM algorithms. This indicates the activation function’s critical role in determining model accuracy.
- Training algorithm: the consistently superior performance of the Levenberg–Marquardt algorithm, particularly when using the hyperbolic tangent’s activation function, resulted in more accurate predictions, smaller errors, and greater adjustments. This was demonstrated by the higher *R*^2^ values and the lower *RMSE* and *MAPE*.
- Overfitting concerns: the phenomenon of overfitting was observed, particularly with higher numbers of neurons in the hidden layer. The best architectures were achieved by employing a hidden layer with 27–32 neurons. The results indicated that it is crucial to avoid overly complicated architectures, since the models with excessive numbers of neurons tended to perform extraordinarily well during the training but faltered during the testing in terms of predictive ability.
- Topologies’ effectiveness: across different training methods and activation functions, the topologies with one hidden layer and with 28–32 neurons demonstrated superior performance. For instance, the [6-28-1] utilizing the hyperbolic tangent function under the LM algorithm showed the best accuracy, with the highest *R*^2^ (0.909). This topology is superior due to the balance between its performances in the training and testing phases, illustrating its potential for accurate real-world predictions. The performance of an ANN can be affected by the quality, size, and representativeness of the dataset used for its training. If the dataset is not sufficiently diverse, it may not be able to capture the full range of conditions that exist in real-world scenarios.

In addition, considering the database heterogeneity used to train the ANN and the high prediction accuracy of the ANN-based model developed, the artificial neural networks were demonstrated to be efficient tools for predicting concrete’s compressive strength, assisting in the mix design of recycled aggregate concrete.

This study’s reliance on a particular dataset might introduce biases or limitations due to the variability in the data used. The ANN’s performance might be influenced by the quality, size, or representativeness of the dataset. Additionally, if the dataset used for the training is not sufficiently diverse, it might not capture the full range of conditions present in real-world scenarios. Thus, in a future work, the quality and size of the dataset will be evaluated regarding their influence on the prediction accuracy and the ANN’s learning capacity.

## Figures and Tables

**Table 1:** Data on statistical parameters.

Feature	Minimum	Maximum	Standard Deviation	Average	Q1 (25%)	Q2 (50%)	Q3 (75%)
CC (kg/m^3^)	171.60	572.00	73.38	377.73	320.00	380.00	423.50
FAG/C	0.57	4.36	0.64	1.94	1.51	1.80	2.24
CAG/C	0.57	6.00	0.71	2.82	2.40	2.70	3.22
RCA	0.00	1.00	0.38	0.36	0.10	0.20	0.61
ADMX	0.00	0.37	0.10	0.12	0.02	0.05	0.07
W/B	0.22	0.76	0.12	0.47	0.40	0.49	0.54
CS (MPa)	21.50	69.80	11.31	41.77	33.30	41.55	48.40

## Data Availability

The data presented in this study are available on request from the corresponding author.
